# A comparison of disease susceptibility and innate immune response between diploid and triploid Atlantic salmon (*Salmo salar*) siblings following experimental infection with *Neoparamoeba perurans*, causative agent of amoebic gill disease

**DOI:** 10.1017/S0031182017000622

**Published:** 2017-05-11

**Authors:** LYNN CHALMERS, JOHN F. TAYLOR, WILLIAM ROY, ANDREW C. PRESTON, HERVE MIGAUD, ALEXANDRA ADAMS

**Affiliations:** Institute of Aquaculture, University of Stirling, Stirling, FK9 4LA, UK

**Keywords:** triploid, amoebic gill disease, AGD, cohabitation, immune response

## Abstract

Few studies have focussed on the health and immunity of triploid Atlantic salmon and therefore much is still unknown about their response to commercially significant pathogens. This is important if triploid stocks are to be considered for full-scale commercial production. This study aimed to investigate and compare the response of triploid and diploid Atlantic salmon to an experimental challenge with *Neoparamoeba perurans*, causative agent of amoebic gill disease (AGD). This disease is economically significant for the aquaculture industry. The results indicated that ploidy had no significant effect on gross gill score or gill filaments affected, while infection and time had significant effects. Ploidy, infection and time did not affect complement or anti-protease activities. Ploidy had a significant effect on lysozyme activity at 21 days post-infection (while infection and time did not), although activity was within the ranges previously recorded for salmonids. Stock did not significantly affect any of the parameters measured. Based on the study results, it can be suggested that ploidy does not affect the manifestation or severity of AGD pathology or the serum innate immune response. Additionally, the serum immune response of diploid and triploid Atlantic salmon may not be significantly affected by amoebic gill disease.

## INTRODUCTION

Sexual maturation in fish causes the transfer of energy from normal somatic growth to gonadal development. This can have adverse effects on body growth rates and flesh quality, and may increase incidences of disease and mortality (Ojolick *et al.* 1995; Leclercq *et al.* 2011). Sexually mature fish that escape from production sites also have the potential to interact with wild fish, impacting on the genetics and fitness of wild populations (Glover *et al.* 2013). As such, sexual maturation is of serious concern for the salmonid aquaculture industry and work continues to derive a solution to these problems. Triploidy is the only commercially available and acceptable means of achieving sterility in fish, and is increasingly being used as a method to control sexual maturation (Oppedal *et al.* 2003; Taylor *et al.* 2011). Triploidy can be readily induced through the application of a hydrostatic or temperature ‘shock’ to newly-fertilized eggs and the process has been optimized for several commercially important species in aquaculture including Atlantic salmon (*Salmo salar*), rainbow trout (*Oncorhynchus mykiss*), brown trout (*Salmo trutta*), turbot (*Scophthalmus maximus*) and grass carp (*Ctenopharyngodon idella*) (Tiwary *et al.* 2004; Maxime, 2008; Piferrer *et al.* 2009; Preston *et al.* 2013). This ‘shock’ treatment prevents second meiotic division and causes the retention of the second polar body, which results in three sets of chromosomes rather than two, and in turn sterility in triploid fish (Tiwary *et al.* 2004; Maxime, 2008). However, despite the clear advantages of being sterile, if triploid Atlantic salmon are to be considered for commercial production, they must perform as well as diploids in all aspects of their biology and physiology. Recent studies have continued to explore and elucidate the physiology and performance of triploid salmon. These results show that the commonly reported problems of deformity, poor survival and reduced growth in triploids can be addressed through refined husbandry, feeds and management, thus further supporting the application of triploids in the aquaculture industry (Burke *et al.* 2010; Fjelldal and Hansen, 2010; Leclercq *et al.* 2011; Taylor *et al.* 2011, 2012). However, very few studies have focussed on triploid Atlantic salmon health and immunity.

Understanding how triploid fish cope with health challenges is an important milestone to characterize their robustness, especially as disease and subsequent health issues continue to restrict the development and success of the aquaculture industry (Weber *et al.* 2013). Anecdotal evidence suggests no differences in mortality of triploid salmon compared with diploid siblings when challenged with disease in commercial settings although no scientific assessments were carried out. A study by Frenzl *et al.* (2014) showed similar infection levels between ploidy when challenged with parasitic sea lice, *Lepeophtheirus salmonis,* in both experimental and natural challenge conditions. In contrast, higher *Gyrodactylus salaris* infection levels in triploids were reported by Ozerov *et al.* (2010). Additionally, triploid goldfish (*Carassius auratus*) showed higher loads of *Metagonnimus* sp. than diploids (Hakoyama *et al.* 2001) and findings from rainbow trout showed triploids were more susceptible to infection by *Ergasilus sieboldin* (Tildesley, 2008). However, with an overall lack of conclusive data, the response of triploid Atlantic salmon to economically significant parasites remains to be fully elucidated.

One such parasite is *Neoparamoeba perurans*, a free-living, marine amoebae and the causative agent of amoebic gill disease (AGD) (Young *et al.* 2008*a*; Crosbie *et al.* 2012). AGD has represented a significant problem for the marine salmonid industry since the mid-1980's, particularly in Tasmania, and has now become a persistent problem for a growing number of countries including Ireland, Scotland and Norway (Oldham *et al.* 2016). Clinical signs of AGD include respiratory distress, flared opercula and lethargy, and macroscopic manifestation of white mucoid patches on the gills (Munday, 1986; Munday *et al.* 1990). Histological examination shows profound changes to gill architecture including extensive hyperplasia and lamellar fusion (Munday, 1986; Roubal *et al.* 1989; Adams and Nowak, 2003). It is now recognized that AGD is the most significant disease caused by gill parasites in terms of economic impact (Shinn *et al.* 2015). While numerous studies have been undertaken to elucidate the interaction of *N. perurans* with the host fish immune system (Gross *et al.* 2005; Pennacchi *et al.* 2014, 2016), further research is required to fully explain the overall immune response of Atlantic salmon to AGD. This is particularly important in relation to triploids with a previous study showing reduced survival in triploids when challenged with *N. perurans* but fewer gill lesions than diploids and no ploidy differences in immune response (Powell *et al.* 2008).

The aim of this study was therefore to investigate and compare the response of triploid and diploid Atlantic salmon to an experimental cohabitation challenge by *N. perurans* and to assess their susceptibility and immune response to this parasite.

## MATERIALS AND METHODS

### Ethical approval

Experimental procedures were approved by the Animal Welfare and Ethical Review Body (AWERB) of the University of Stirling and were completed under UK Government Home Office project licence 60/4189. The euthanisation of fish for sampling was carried out according to the UK Government Home Office Schedule 1 regulations.

### Fish stock & history

Fish used in this study were obtained from two commercial breeding companies (Stock A and Stock B) and supplied as eyed ova (Stock A: 372°D, 21 December 2012; Stock B: 380°D, 4 January 2013) to the University of Stirling Freshwater Research Facility (56°N 4°W). Triploidy was induced at both company sites using the same protocol, whereby post-fertilization, half of each egg batch (Stock A, 26 unrelated dams and 6 sires; Stock B, 20 unrelated dams and 5 sires) was exposed to a hydrostatic pressure shock of 655 bar applied for 6·25 min, 37·5 min post fertilization at 8 °C. Ova (3 tanks per ploidy per stock with 2500 ova per tank) were incubated in complete darkness at 7·1 ± 0·3 °C until hatching, where temperature was gradually increased to 10 °C for first feeding (Stock A: 25 February 2013; Stock B: 4 March 2013). At first feeding, fish were reared under constant light and fed a commercial diet (Diploids – Inicio Plus; Triploids – Inicio-TriX, BioMar UK), distributed by automatic feeders (Arvo-Tec Oy, Finland). Specific feeding rates (% tank biomass per day) were adjusted automatically according to predicted growth and daily temperature, and pellet size (0·5 to 2 mm) increased with fish size. From August 2013, fish were maintained under ambient temperature (min: 3 °C, max: 15 °C) and photoperiod to produce S1+ smolts for sea transfer on 22 April 2014 (Marine Environmental Research Laboratory (MERL), Institute of Aquaculture, Machrihanish, UK, 55·4°N 5·7°W). Fish were vaccinated in November 2013 with Alphaject 2·2 (PHARMAQ, UK). Mortality between first feeding and sea transfer was 1·18 and 1·99% in Stock A, and 2·8 and 3·5% in Stock B, respectively for diploids and triploids. Following sea transfer, fish were maintained under ambient temperature (min: 9 °C, max: 11 °C) in 1 m stock tanks (400 L; 1 L kg biomass^−1^ min^−1^ flow rate) with aeration provided by air stones for 24 days until allocation to challenge tanks. During this period, mortality was 1·32% for diploids and 1·79% for triploids in Stock A, and 4·9% for diploids and 0·3% triploids for Stock B.

To verify ploidy status in each stock, smears were prepared from blood collected by tail ablation from euthanised fish at 5 g (100 per ploidy). After air drying, slides were fixed in 100% methanol and then placed into 6% Giemsa stain (6 mL Giemsa in 94 mL distilled water) for 10 min. Erythrocyte length and diameter were measured at 40 × magnification using image capture software (Image-Pro Premier, MediaCybernetics, Rockville, USA). All erythrocytes were numbered then selected using a random number generator. A total of 20 randomly chosen nuclei per slide were measured to the nearest 0·01 *µ*m. Diploid control groups had significantly smaller erythrocyte nuclear lengths with no overlaps with the pressure shock triploid groups (2N 6·8–7·7 *µ*m; 3N 9·0–10·2 *µ*m) confirming that fish subjected to hydrostatic pressure shock were triploids.

### Cohabitation challenge

On 12 May 2014, fish were randomly allocated into twelve 1 m tanks (400 L; 1 L kg biomass^−1^ min^−1^ flow rate), with the ploidies held separately (6 tanks ploidy^−1^). Thirty fish from each Stock (Stock A: 2N 93·2 ± 8·9 g, 3N 85·8 ± 9·8 g; Stock B: 2N 98·8 ± 13·6 g, 3N 83·1 ± 13·4 g) were added to appropriate ploidy tanks (60 fish tank^−1^) before being randomly allocated to a challenge group in triplicate: diploid uninfected and infected; triploid uninfected and infected (3 tanks group^−1^). Stock B fish were fin-clipped to distinguish them from Stock A. Fish were maintained under ambient temperature (min: 11 °C, max 13 °C). The challenge facility was flow-through in configuration, supplied with 100 *µ*m filtered natural seawater. Water quality was checked and maintained at >7 mg L^−1^ dissolved oxygen, pH 8·3, <0·25 mg L^−1^ NH_3_-N, <0·15 mg L^−1^ NO_2_-N and <5 mg L^−1^ NO_3_-N.

The cohabitation challenge was undertaken according to methods developed at the Institute of Aquaculture, Stirling, Scotland. Challenge cohabitants were produced using a stock of infected Atlantic salmon held at the MERL facility as part of an *in vivo* amoebae culture. Four of these pre-infected fish were added to a separate stock of 40 naïve Atlantic salmon smolts. Gills were grossly assessed until the appropriate gill score for cohabitation infection (approximately 1·5–2 gill score) was achieved. The cohabitants (seeder fish) were adipose fin-clipped, marked with panjet (0·0652 g alcian blue mL^−1^, Sigma-Aldrich, UK) and added to the appropriate challenge groups (6 cohabitants tank^−1^). A group of uninfected cohabitants were also produced using the same method, with 4 uninfected Atlantic salmon added to another stock of 40 naïve smolts. No clinical pathology was observed in uninfected seeders after 2 weeks. Sampling occurred at 7, 14, 21 and 28 days post infection (dpi), after which the challenge was terminated. At each sampling point, 5 fish per stock per tank were removed from the tanks and culled by lethal anaesthesia (10% Benzocaine, Sigma-Aldrich, UK) before being sampled. Gills were visually assessed and scored for gill lesion severity according to Taylor *et al.* (2009). Blood was then sampled from the caudal vein using a non-heparinised needle and syringe. Blood samples were kept overnight at 4 °C before being centrifuged at 3000 ***g*** for 10 min to collect serum. Finally, the left 2nd gill arch was excised into paraformaldehyde for histological processing.

### Histopathology

After 24 h fixation in paraformaldehyde, gills were transferred into 70% ethanol. Gills were dehydrated (Thermo Shandon Citadel 2000), embedded in paraffin wax, sectioned at 5 *µ*m and stained with haematoxylin and eosin. To assess changes in pathology severity, gill sections from 7 and 28 dpi were analysed. The total number of filaments on each gill section were counted along with the number of filaments presenting AGD-associated lesions. These lesions were defined according to Adams *et al*. (2009) with hyperplastic fusion of the secondary lamellae in close proximity to *N. perurans* trophozoites. The percentage of filaments affected by AGD lesions was subsequently calculated.

### Histochemistry

The second left gill arch from all fish, fixed and processed as above, were stained for the detection of mucous cells using a combined alcian blue-PAS technique according to Mowry (1956), with modifications. Briefly, sections were de-waxed, rehydrated and immersed in alcian blue solution (pH 2·5) for 5 min. The residual stain was removed by washing in water. Sections were oxidized in 1% (aq) periodic acid (5 min), washed (5 min) and immersed in Schiff's reagent (20 min). Finally, sections were washed in running tap water (10 min) and counterstained with haematoxylin Z (2 min) before being washed, dehydrated, cleared and mounted. To assess changes in mucous cell populations, mucous cells from fish at 7 and 28 dpi were counted and measured. Sections were first scanned using a slide scanner (Pannoramic 250 Flash III, 3DHISTECH Ltd, UK). To then assess changes in cell number, mucous cells were counted in five regions across a gill section image using ImageJ (Maryland, USA). Mucous cell size was also assessed by measuring the diameter of 20 random cells on the gill arch (CaseViewer, 3DHISTECH Ltd, UK)

### Immunohistochemistry

Chloride cells were targeted for immunohistochemical identification according to Adams and Marìn de Mateo (1994). Chloride cell staining used gill tissue fixed as previously described. Sections were de-waxed in xylene (10 min) and rehydrated through a graded ethanol series (100% for 5 min and 70% for 3 min). Sections were then blocked for endogenous peroxidase activity (3% H_2_O_2_ in methanol – 20 min), washed with PBS (5 min) and incubated with 10% normal goat serum (20 min) to block non-specific binding sites. Sections were blotted dry and incubated overnight in a humid chamber with a mouse monoclonal antibody to Na+/K+-ATPase (1:200, IgGα5, Developmental Studies Hybridoma Bank, Department of Biological Sciences, University of Iowa, Iowa, USA). Sections were washed with PBS and incubated (1 h, room temperature) with 1:200 goat anti-mouse IgG (A4416, Sigma, USA) before being washed again. ImmPACT DAB Peroxidase (HRP) Substrate (SK-4105; Vector Laboratories, Burlingame) was applied as per manufacturer instructions and sections were incubated for 5 min. Slides were then immersed in tap water (2–5 min) to stop the reaction, counterstained with haematoxylin Z (3 min), dehydrated, cleared and mounted.

### Lysozyme

Lysozyme activity in serum samples was measured turbidimetrically according to Morgan *et al.* (2008). Following completion of the assay, the reduction in absorbance at 540 nm was measured at 1 min intervals for 5 min using a microplate reader (Synergy HT, BioTek Instruments, USA) and the Gen5 Data Analysis Software. One unit of lysozyme activity is defined as the amount of sample causing a decrease in absorbance at 0·001 min^−1^. Activity was expressed as units min^−1^ ml^−1^.

### Complement activity

Spontaneous haemolytic activity was determined by adapting the method described by Langston *et al.* (2001). Briefly, serum samples in duplicate were doubly diluted in 0·1% gelatine veronal buffer (GVB) (1 complement fixation tablet (Oxoid, UK), 0·1 g gelatine) in a U-well microplate (final volume 25 *µ*L well^−1^). A 5% sheep red blood cell (SRBC) suspension was then added to all wells (10 *µ*L well^−1^). Positive controls (100% lysis) of 0·1% anhydrous Na_2_CO_3_ plus SRBC, and negative controls (0% lysis) of GVB plus SRBC were also added to the microplates. All samples were incubated for 90 min at room temperature (RT) with constant shaking then the reaction was stopped by adding GVB with 20 mm EDTA (Sigma, UK) (140 *µ*L well^−1^). The microplates were centrifuged at 750 ***g*** for 6 min before supernatants were transferred into flat-well microplates (100 *µ*L well^−1^). The absorbance was measured at 450 nm. The percentage lysis for each sample and dilution was calculated using control values. The dilution that would produce 50% lysis was then determined by PROBIT analysis and the reciprocal expressed as spontaneous haemolysis (SH50%, units mL^−1^).

### Anti-protease activity

Detection of anti-protease activity was undertaken according to the methods of Ellis (1990) and Thompson *et al.* (1995), with modifications. A 100 *µ*L mL^−1^ trypsin solution was prepared by adding 1 mL of 25 mg mL^−1^ of trypsin stock solution to 249 mL 0·1 M Tris.HCl (pH 8·2). In round-bottomed 96-well microplates, serum samples were diluted two-fold in Tris.HCl buffer (20 *µ*L well^−1^), followed by 60 *µ*L of trypsin solution. Plates were then incubated for 5 min at RT. Finally, chromogen solution in distilled water (0·1% Nα-Benzoyl-L-arginine 4-nitroanilide hydrochloride (BAPNA)) was added to each well. Controls were also added to each microplate: As a zero reference, only BAPNA solution and Tris.HCl buffer were added to wells while wells containing BAPNA solution, Tris.HCl buffer and trypsin solution were used as positive controls. All plates were incubated at RT for 30 min before being centrifuged at 750 ***g*** for 6 min. One hundred *μ*L from each well was transferred into new flat-bottomed microplates and then absorbance (410 nm) was measured using a microplate reader. Trypsin inhibition (%) was then calculated as ((positive control OD – sample OD)/positive control OD) × 100.

### Statistical analysis

Minitab software version 16 (Minitab Inc., Pennsylvania) was used in this study to perform basic descriptive statistics and comparisons using a significance level of 5% (*P* = 0·05). Prior to analysis, datasets were checked for normality using the Anderson-Darling test. Where necessary, log transformations were performed to normalize the data. Post-hoc analyses were carried out using Tukey's multiple comparison tests with values considered significantly different at *P*-values <0·05. Non-parametric Kruskal–Wallis test was used to assess gross gill score. General linear model manipulated to four-way analysis of variance (ANOVA) was used to analyse histopathology severity, mucous cell parameters, lysozyme, complement and anti-protease activities, with ploidy, infection, time (dpi) and stock considered fixed factors. Three way-ANOVA was also used to assess the previously mentioned parameters with ploidy, infection and, time (dpi) as fixed factors. Further three-way ANOVA was used to analyse total mortality (%) with ploidy, infection and stock as fixed factors. In addition, survival was analysed by Kaplan–Meier survival analysis, with differences between groups determined by log-rank tests (MedCalc, MedCalc Software, Belgium).

## RESULTS

### Challenge mortality

For Stock A, total mortality was 6·7 ± 1·9, 3·3 ± 1·9, 13·3 ± 10 and 8·9 ± 2·2% for the diploid uninfected, triploid uninfected, diploid infected and triploid infected groups respectively. In the diploid uninfected, triploid uninfected, diploid infected and triploid infected groups respectively groups from Stock B, total mortality for the challenge was 3·3 ± 3·3, 7·8 ± 2·9, 11·1 ± 2·9 and 7·8 ± 4·8%, respectively. Statistical analysis found no significant effect of ploidy, infection or stock on total mortality. Kaplan–Meier survival analysis was also performed and similar survival probability was exhibited in all groups from both stocks (0·92 ± 0·03 to 0·99 ± 0·01). No significant effect of stock, ploidy or infection was observed (data not shown).

### Gill scores

**Fig. 1.**
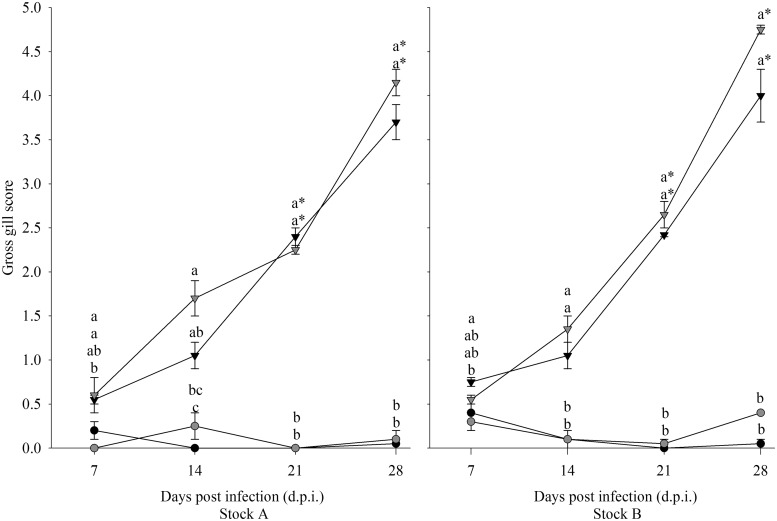
Progression of gill scores over time in infected (triangle) and uninfected (circle), diploid (black) and triploid (grey) Atlantic salmon from Stock A and B. Statistically significant differences between groups at each time point are indicated by different lowercase letters. Significant time differences relative to 7 dpi are indicated by asterisk (*).

Gill scores of all diploid and triploid infected groups increased over time, while those of uninfected groups remained low (Fig. 1). Both stock and ploidy did not have a significant effect on gill score. For both stocks, time and infection had a significant effect. Gill scores in the infected groups from 14 dpi onwards were significantly higher than at 7 dpi, and all infected groups had significantly higher gill scores compared with their respective uninfected group.

### Histopathological severity

**Fig. 2.**
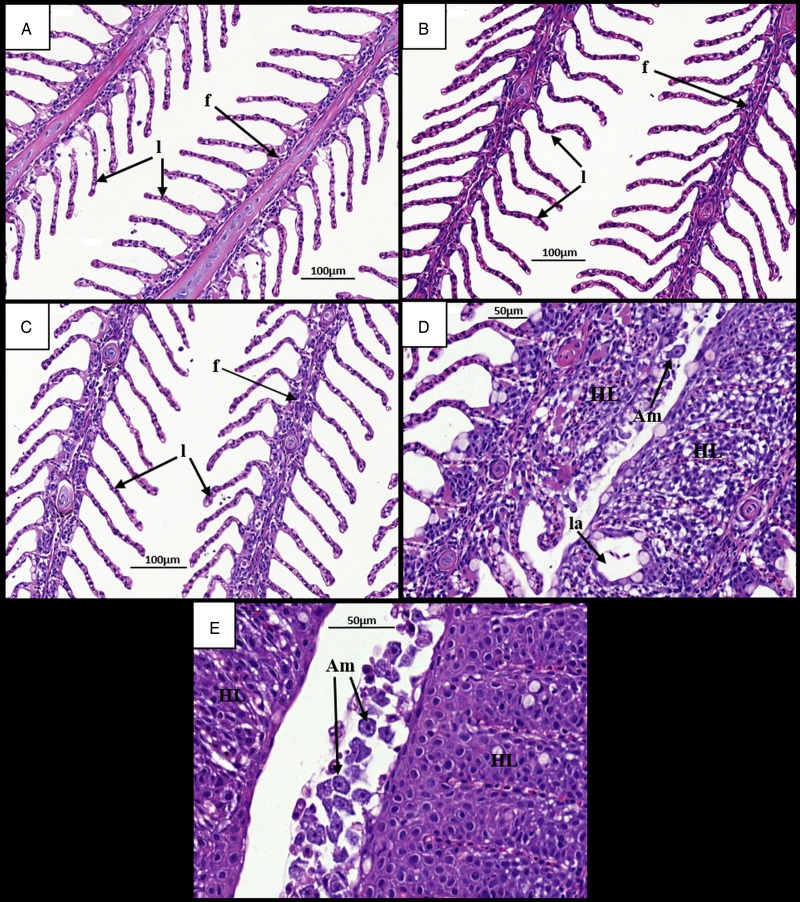
Time changes in histological pathology in the gills of infected and uninfected diploid Atlantic salmon. (A) Uninfected fish at 7 dpi. Individual filaments (f) and lamellae (l) visible. (B) Infected fish at 7 dpi. (C) Uninfected fish at 28 dpi. Individual filaments and lamellae remain visible. (D) Infected fish at 28 dpi. Hyperplastic AGD lesions (HL) clearly visible with lamellar fusion causing lacunae formation (la). Amoeba (Am) visible next to lesion. (E) Infected fish at 28 dpi. Numerous amoebae associated with hyperplastic gill tissue. AGD, amoebic gill disease.

**Fig. 3.**
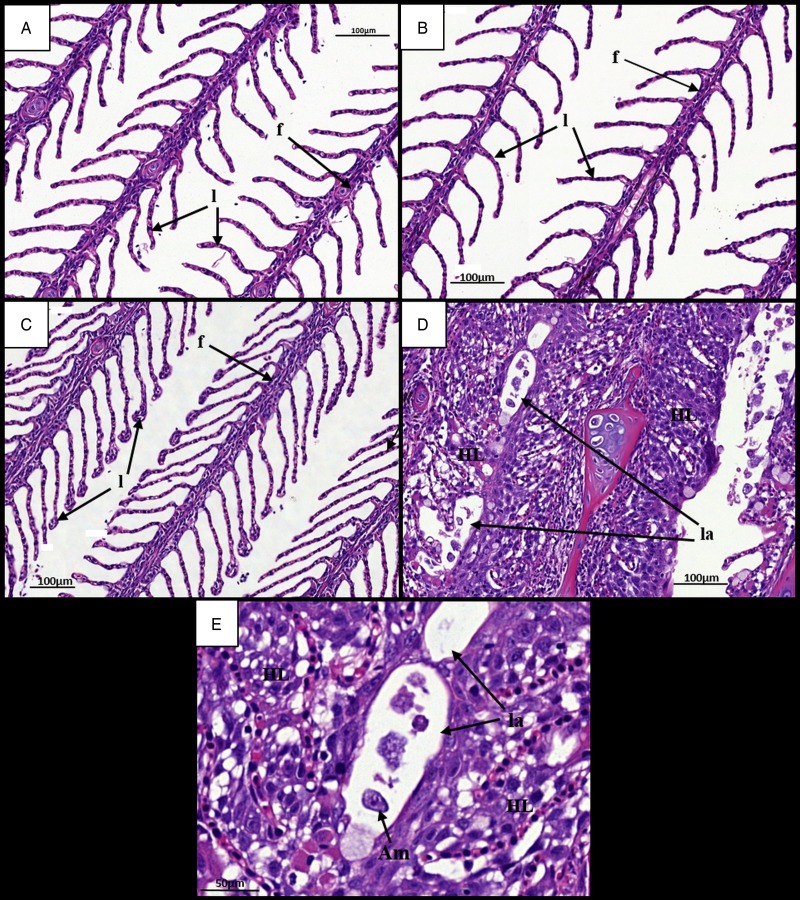
Time changes in histological pathology in the gills of infected and uninfected triploid Atlantic salmon. (A) Uninfected fish at 7 dpi. Individual filaments (f) and lamellae (l) visible. (B) Infected fish at 7 dpi. (C) Uninfected fish at 28 dpi. Individual filaments and lamellae remain visible. (D) Infected fish at 28 dpi. Hyperplastic AGD lesions (HL) visible with lamellar fusion causing lacunae formation (la). (E) Infected diploid at 28 dpi. Single amoeba contained within a gill lacunae. AGD, amoebic gill disease.

Gills at 7 dpi appeared normal in all ploidy groups from both stocks, with individual filaments and lamellae clearly visible (Figs 2A, B and 3A, B). Analysis of diploid and triploid gills at 28 dpi revealed that, while the gill structure of uninfected fish remained unchanged over time (Figs 2C and 3C), noticeable structural changes had occurred in the infected groups. In all diploid and triploid infected groups, severe hyperplasia and hypertrophy were clearly evident as well as lamellar fusion resulting in lacunae formation (Figs 2D, E and 3D, E). Within the lacunae, amoebae were often visible (Fig. 3E). To further support visual analyses, the percentage of gills showing AGD-associated lesions was analysed (Table 1). Time had a significant effect in the infected groups with significantly higher percent of affected gill filaments recorded at 28 dpi (Stock A: 2N 38·3, 3N 49·8; Stock B: 2N 34·6, 3N 41·8%) than at 7 dpi (Stock A: 2N 0, 3N 0; Stock B: 2N 0, 3N 0%) (Table 1). Infection significantly affected both stocks at 28 dpi, with the infected groups showing higher affected gill filaments (%) than their respective uninfected group. In addition, ploidy had a significant effect on the Stock A infected groups at 28 dpi, with triploids showing higher affected filaments (%) than their diploid counterparts.

**Table 1.** Comparison of AGD affected filaments (%) (mean ± s.e.m.) in uninfected and infected diploid (2N) and triploid (3N) Atlantic salmon from Stocks A and B at 7 and 28 days post-infection (dpi)

Time (dpi)	2N uninfected	2N infected	3N uninfected	3N infected
Stock A				
7	0 ± 0^c^	0 ± 0^c^	0 ± 0^c^	0 ± 0^c^
28	0 ± 0^c^	38·3 ± 4·5^b^	0 ± 0^c^	49·8 ± 1·3^a^
Stock B				
7	0 ± 0^b^	0 ± 0^b^	0 ± 0^b^	0 ± 0^b^
28	0 ± 0^b^	34·6 ± 1·6^a^	0 ± 0^b^	41·8 ± 6·2^a^

Lowercase superscripts denote significant differences within each Stock.

### Mucous cell counts

**Fig. 4.**
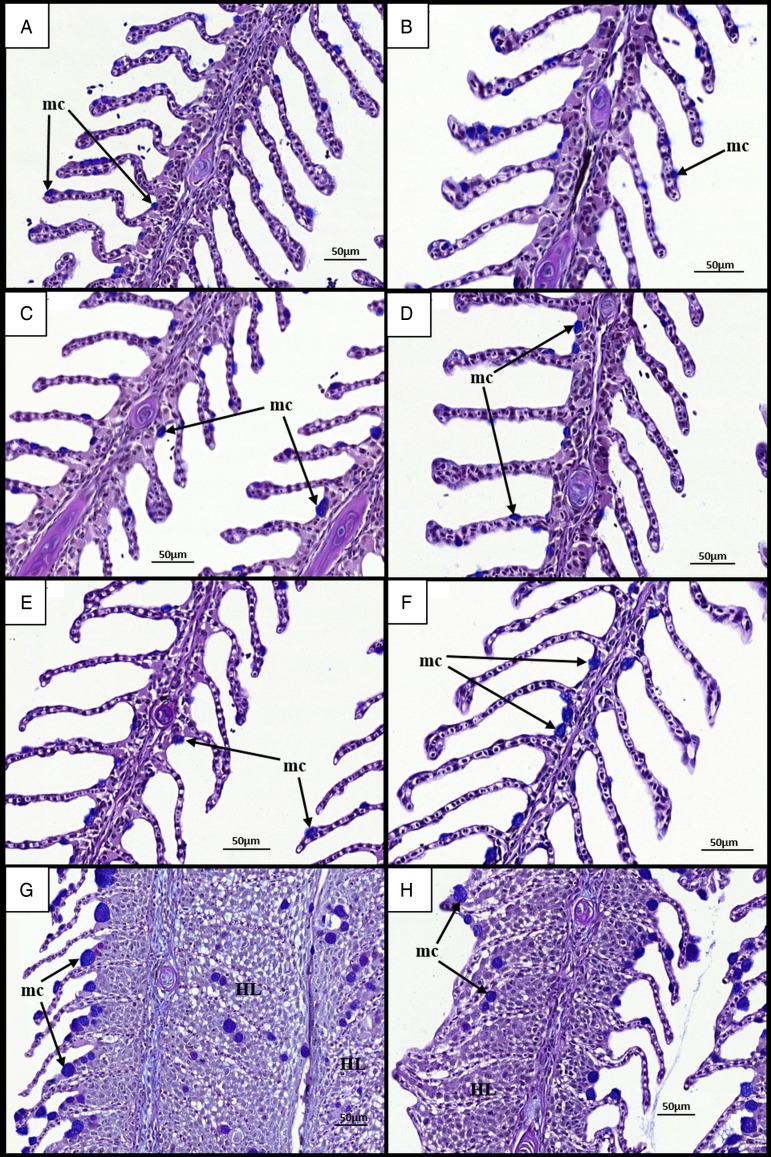
Time changes in mucous cells in the gills of infected and uninfected diploid and triploid Atlantic salmon. (A) Uninfected diploid at 7 dpi. Individual filament (f) and lamellae (l) visible. Small mucous cells (mc) observed on lamellae. (B) Uninfected triploid at 7 dpi. (C) Infected diploid at 7 dpi. (D) Infected triploid at 7 dpi. (E) Uninfected diploid at 28 dpi. Mucous cell remain unchanged (F) Uninfected triploid at 28 dpi. (G) Infected diploid at 28 dpi. Hypertrophied mucous cells visible on and around hyperplastic gill lesions (HL). (H) Infected triploid at 28 dpi.

Histological examination of gill mucous cells at 7 dpi revealed small mucous cells in low numbers near or at the bases of secondary lamellae in all groups (Fig. 4A–D). At 28 dpi, analysis of diploid and triploid uninfected gills revealed little change in the appearance and position of mucous cells (Fig. 4E and F). However, in all infected groups, noticeable changes had occurred in the mucous cell populations (Fig. 4G and H). Mucous cells were found distributed along the entire length of the lamellae. Additionally, there appeared to be an increase in mucous cell number and diameter. In terms of cell number, no significant effect of stock, ploidy, nor time was observed (Table 2). In the Stock A groups at 28 dpi, there was a significant effect of infection upon diploid and triploid groups, exhibiting significantly higher number than their respective uninfected group. For cell diameter, stock did not have a significant effect. Time had a significant effect on cell diameter in all infected groups and in the Stock B triploid uninfected group, with greater cell diameter recorded at 28  than at 7 dpi. Infection was found to have a significant effect on cell diameter at 28 dpi, with significantly higher size recorded in the infected groups compared with their respective uninfected group (Table 2). Ploidy had a significant effect on the uninfected groups of Stock A, with larger cell diameter recorded in triploids.

**Table 2.** Comparison of mucous cell number and diameter (*μ*m) (mean ± s.e.m.) in uninfected and infected diploid (2N) and triploid (3N) Atlantic salmon from Stock A and B at 7 and 28 days post-infection (dpi)

	Mucous cell counts				Mucous cell diameter (*μ*m)			
Time (dpi)	2N uninfected	2N infected	3N uninfected	3N infected	2N uninfected	2N infected	3N uninfected	3N infected
Stock A								
7	256·0 ± 36·0	288·0 ± 38·0	249·0 ± 21·0	281·0 ± 35·0	8·49 ± 0·07^b^	9·54 ± 0·19^ab^	10·71 ± 0·36^a^	9·93 ± 0·42^ab^
28	181·0 ± 44·0^b^	343·0 ± 2·0^a^	187 ± 3·0^b^	329·0 ± 19·0^a^	10·12 ± 1·00^b^	15·79 ± 0·39^a^*	11·64 ± 0·21^ab^	15·84 ± 1·16^a^*
Stock B								
7	204·0 ± 31·0	259·0 ± 8·0	282·0 ± 24·0	219·0 ± 41·0	7·94 ± 0·32	7·81 ± 0·28	9·17 ± 0·05	9·20 ± 0·29
28	217·0 ± 18·0	356·0 ± 38·0	195·0 ± 19·0	354·0 ± 67·0	10·35 ± 0·22^c^	15·43 ± 0·74^ab^*	13·08 ± 0·6^bc^	17·52 ± 0·74^a^*

Lowercase superscripts denotes significant differences between groups at each time point within each Stock.

Asterisk (*) denotes significant difference between 7 and 28 dpi for each group within each Stock.

### Chloride cells

**Fig. 5.**
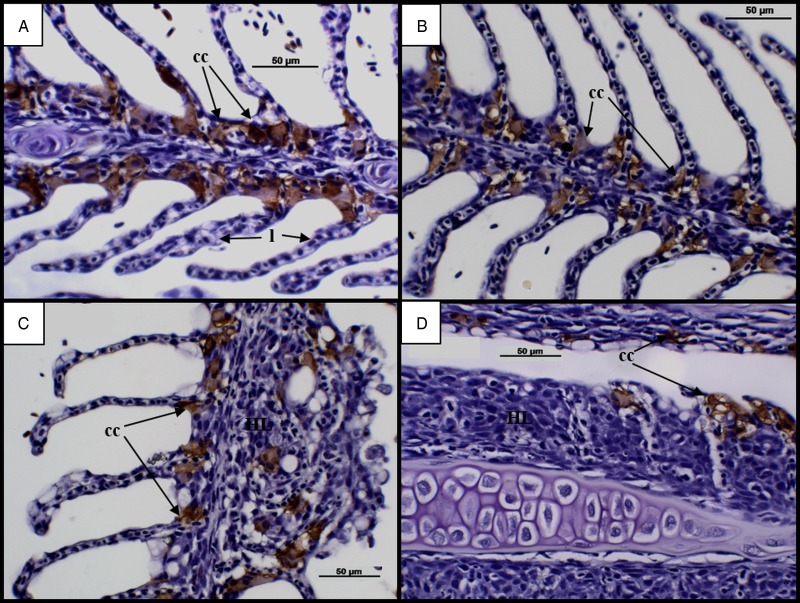
Time changes in chloride cells in infected diploid and triploid Atlantic salmon. (A) Diploid at 7 dpi. Chloride cells (cc) visible in abundance along bases of lamellae (l). (B) Triploid at 7 dpi. (C) Diploid at 28 d. Chloride cells found at the tips of lamellae. Chloride cells absent along advanced hyperplastic AGD lesions (HL). (D) Triploid at 28 dpi.  AGD, amoebic gill disease.

An assessment of chloride cells in infected groups at 7 dpi revealed chloride cells in abundance along the bases of secondary lamellae (Fig. 5A and B). Time dependent changes became evident at 28 dpi, with cells observed towards the tips of lamellae and an apparent reduction in cell number along advanced AGD lesions (Fig. 5C and D).

### Immune response

**Fig. 6.**
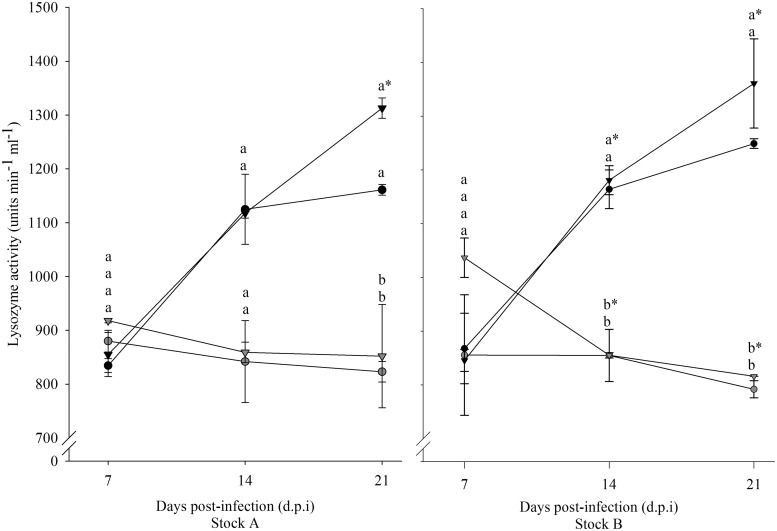
Lysozyme activity (unit min^−1^ mL^−1^) over time in infected (triangle) and uninfected (circle), diploid (black) and triploid (grey) Atlantic salmon from Stocks A and B. Statistically significant differences between groups at each time point are indicated by different lowercase letters. Significant time differences relative to 7 dpi are indicated by asterisk (*).

Over time lysozyme activity increased in the infected and uninfected diploid groups of both stocks while it decreased in all the triploid groups (Fig. 6). Stock and infection did not have a significant effect on lysozyme activity. Time had a significant effect on the infected diploid groups of Stock A and B, with higher lysozyme activity recorded at 21 dpi compared with 7 dpi. Additionally, significantly lower lysozyme at 21 dpi than 7 dpi was recorded for the Stock B infected triploid group. Ploidy had a significant effect on lysozyme activity. In Stock A, significantly higher activity was recorded for both diploid groups at 21 dpi., while for Stock B, both diploid groups showed significantly higher activity than the triploid groups at 14 and 21 dpi.

Variable levels of complement activity (SCH50%, units ml^−1^) were recorded in all groups from both stocks (A 29 ± 4·99; B 26 ± 5·30) but neither stock, ploidy, infection nor time had a significant effect on complement activity (data not shown).

Similar levels of anti-protease activity (trypsin inhibition, %) were exhibited by all groups from both stocks (A 61 ± 5·30; B 57 ± 6·15) and no significant effect of stock, ploidy, infection or time were observed (data not shown).

## DISCUSSION

As triploid Atlantic salmon are now being used more extensively, elucidating how they cope with AGD is important in determining their robustness and therefore their suitability for commercial aquaculture practices (Weber *et al.* 2013). This study compared the response of diploid and triploid Atlantic salmon to infection with *Neoparamoeba perurans*, the causative agent of amoebic gill disease.

Throughout the 28 day challenge, mortality remained low in all groups. Ploidy did not have a significant effect on challenge mortality, and this concurs with findings of a Scottish field based study in which sibling groups of diploid and triploid Atlantic salmon experienced a natural AGD challenge (temp range 11–15 °C, comparable with this study), yet mortality was comparable between ploidy (Smedley *et al*. 2016). However, these results are in contrast to an experimental infection study with *N. perurans* performed in Tasmania in which triploids were shown to have lower survival than diploids (Powell *et al*. 2008). Unfortunately, details of challenge model were not described in Powell *et al*. (2008), and as such contrasting results between this study and ours may arise from differences in challenge severity, water temperature, other environmental factors, origin of fish, or location. Infection did not significantly affect mortality. While infected fish would have been expected to experience higher mortalities, considering the levels of mortality associated with AGD outbreaks (Oldham *et al.* 2016), it is not possible to make study comparisons at this time due to the limited experimental cohabitation or bath challenge studies presenting mortality data. As such, studies should be planned to fully assess the occurrence and progression of mortalities in response to experimental AGD.

A key feature of AGD pathology is the occurrence of white mucoid patches on the gills, and these are used to grossly assess and score infection level (Taylor *et al.* 2009). In this study, gross gill scores increased over time in the diploid and triploid infected groups while they remained consistently low in the uninfected groups. This is consistent with previously published 28 day AGD cohabitation studies (Findlay *et al*. 1995; Zilberg and Munday, 2000) as well as bath challenge studies of the same duration (250 amoebae trophozoites L^−1^) (Norte dos Santos *et al.* 2013) or shorter (10 days) with a higher amoebae load (2000 trophozoites L^−1^) (Pennacchi *et al.* 2014). Additionally, non-significant differences in the levels of grossly observable pathology between groups for each time point indicate that the cohabitation challenge utilized provided a consistent approach to inducing AGD under experimental conditions. In the present study, ploidy did not have a significant effect on gill score severity which is consistent with a previous study indicating that ploidy did not affect the severity of infection with sea lice (*L. salmonis*) (Frenzl *et al.* 2014).

In addition to gross gill scores, histological gill examination is also utilized to indicate both pathogen presence and host response (Adams *et al.* 2004; Taylor *et al.* 2009). Taking into account the increase in gross gill score severity over time, it was considered that the gills from 7 and 28 dpi sampling events would show the most severe pathological changes and were therefore chosen for histological processing and examination. Clear alterations to gills were evident in the infected groups. While normal gill structure was observed at 7 dpi, extensive epithelial hyperplasia as well as complete lamellar fusion and lacunae formation was found at 28 dpi. Amoebae were also found associated with areas of the infected gill tissue and within the lacunae. These gill changes are amongst the key pathological features of AGD and are supported by a 19 week study collecting samples from natural AGD outbreaks as well as experimental bath challenges ranging from 10 (250 trophozoites L^−1^) to 16 days (10 000 trophozoites L^−1^) in length (Adams and Nowak, 2003; Leef *et al.* 2005; Peyhan and Powell, 2006). When assessing histopathological changes, the percentage of gill filaments exhibiting AGD-associated lesions was analysed. The percentage of AGD-affected filaments recorded for the uninfected groups remained at 0% for the duration of the study, while it increased significantly in the infected groups. This is consistent with another cohabitation study which found more severe pathology in infected fish at 28 dpi than at 7 dpi (Zilberg and Munday, 2000). In the present study, ploidy had a significant effect on the percentage of AGD-affected gill filaments at 28 dpi in Stock A, with triploids showing a higher percent affected than diploids. In contrast, the opposite was observed by Powell *et al.* (2008) with the diploids showing more affected filaments. In their study, Powell *et al*. (2008) also observed increased mortality in triploids and so it could be suggested that the most severely affected triploids had died before day 28. However, as an assessment of lesion severity in the mortalities was not performed either by Powell *et al*. (2008) or in this study, it is difficult to confirm whether lesion severity had an impact on mortality.

As well as alterations to overall gill structure, infection by *N. perurans* has also been shown to cause changes in mucous cells (Adams and Nowak, 2003; Roberts and Powell, 2003). At 7 dpi, small mucous cells were found distributed along the length of the lamellae. At 28 dpi, while mucous cells remained unchanged in uninfected fish, they were found at the tips of secondary lamellae in the diploid and triploid infected groups. This finding concurs well with previously published research (Roberts and Powell, 2003; Morrison and Nowak, 2008) and is suggestive of a link to the excessive mucus production associated with AGD (Mitchell and Rodger, 2011). Additionally, increases in mucous cell number and size were observed. Infection had a significant effect on the mucous cell numbers of the Stock A groups at 28 dpi, with infected fish showing significantly higher numbers than the uninfected groups. This is consistent with results from previous studies using various infection methods and time-frames, all of which found increased mucous cell numbers in response to infection by *N. perurans* (Zilberg and Munday, 2000; Adams and Nowak, 2003; Roberts and Powell, 2003). Mucous cell diameter increased significantly from 7 to 28 dpi in the infected fish for both stocks and ploidy. As such, larger cells were observed in infected fish at 28 dpi compared with uninfected counterparts. Such hypertrophied mucous cells have been commonly reported with AGD (Adams and Nowak, 2004). The lack of clear and significant differences in mucous cell number and size between ploidy is surprising. Indeed, many studies have reported significantly reduced cell numbers and larger cell volume in triploids as a compensatory mechanism for having three sets of chromosomes. This mechanism has been found in a number of other fish species and cell types, particularly erythrocytes (Small and Benfey, 1987; Stillwell and Benfey, 1996; Budiño *et al.* 2006). However, for both mucous cell counts and measurements, it should be noted that the depth of gill section in which the cells were observed was not constant. It is recognized that this difference may have impacted on the counts and measurements reported and, as such, it is recommended that further work on the impact of AGD on diploid and triploid Atlantic salmon mucous cells be conducted with key attention paid to histological processing and gill section depth.

Along with changes in mucous cells, other AGD associated gill changes include the movement or location of chloride cells (Adams and Nowak, 2003), which was also evident in the current study. It was found that, prior to the appearance of clinical signs in diploid and triploid Atlantic salmon, chloride cells were abundant along the bases of secondary lamellae. By 28 dpi, chloride cells were observed at the tips of secondary lamellae and were missing completely from advanced AGD lesions. These findings are consistent with a 10 day experimental bath challenge as well as studies collecting samples from natural AGD outbreaks over a longer time-frame (Adams and Nowak, 2003; Bermingham and Mulcahy, 2006; Peyhan and Powell, 2006). Fish with extensive AGD lesions have previously been shown to suffer osmoregulatory failure and it has been suggested that reductions in gill chloride cells during AGD may cause increased blood sodium levels (Findlay, 2000; Adams and Nowak, 2003). In contrast, studies have also shown comparable plasma ion and enzyme activity levels in the gills of infected and uninfected fish, suggesting that AGD does not cause osmoregulatory dysfunction (Powell *et al.* 2001, 2008). However, these differences in findings could be attributed to the method of infection used, with studies collecting samples from bath and cohabitation challenges as well as natural in-field challenges. As such, while AGD is known to damage the gills, having the potential to compromise the function of chloride cells (Findlay, 2000; Adams and Nowak, 2003), it is recommended that additional studies on the impact of AGD on chloride cells be carried out to fully clarify these effects.

Previously published data regarding the immune response of Atlantic salmon to AGD have generally focussed gene expression, antibody production and histopathology (Valdenegro-Vega *et al.* 2014). As such, studies have continued to describe and elucidate the host innate and adaptive immune responses when infected with *N. perurans* (Gross *et al.* 2005; Young *et al.* 2008*b*; Valdenegro-Vega *et al.* 2014). In this study, lysozyme activity increased over time in both diploid groups while it decreased slightly in the triploid groups. Although ploidy differences were significant at 21 dpi, the lysozyme activity recorded was found to be in the range previously reported for Atlantic salmon and other salmonids (Saurabh and Sahoo, 2008; Korkea-aho *et al.* 2011; Dick, cited in Nowak *et al.* 2014; Metochis *et al.* 2017). In terms of the infected groups, this significant ploidy difference is in contrast to previous results in which no ploidy differences in serum lysozyme activity were observed (Powell *et al*. 2008). As the study duration was the same, it could be suggested that this difference may be due to the challenge model utilized or parasite load but with no details presented, it is difficult to elucidate. For the uninfected groups, this ploidy difference is also contrasting to previous studies as comparable lysozyme was observed in response to seawater transfer and seasonal changes (Budiño *et al.* 2006; Taylor *et al.* 2007; Tolarová *et al.* 2014). As such, it is unclear why these ploidy differences occurred and it is recommended that studies be carried out to determine ploidy differences in lysozyme in response to AGD. Within ploidy, infection did not have a significant effect on lysozyme activity and this is supported by Gross *et al.* (2005), who found comparable lysozyme activity between infected and uninfected diploid Atlantic salmon throughout an 11 day study. While lysozyme has been shown to change in response to other parasites such as *Ichthyophthirius multifiliis* and *Ceratomyxa shasta* (Alvarez-Pellitero, 2008), the current results suggest that serum lysozyme may not play a major role in AGD as suggested by Gross *et al.* (2005) who proposed that, as lysozyme levels are regulated by peripheral blood leucocytes, infection by *N. perurans* may not stimulate these cells in the serum to elicit a response. In contrast, a study by Cook *et al*. (2012) found a marker encoding a novel g-type lysozyme, the expression of which suggests its involvement in the innate immune response against AGD.

Complement, a group of serum proteins known to assist the killing properties of antibodies (Yano, 1992), has been shown to protect against other parasites including *Crytobia salmonsitica*, *Ichthyophthirius multifiliis* and *Gyrodactylus salaris* (Harris *et al.* 1998; Alvarez-Pellitero, 2008), as well as kill *Gydrodactylus salaris in vitro*. However, limited knowledge is available on its involvement in AGD. Variable patterns of complement activity were observed in this study but with no apparent significant effect of ploidy, infection, time or stock. This is again consistent with previous studies which found no significant change in serum alternative complement activity following infection with *N. perurans* (Powell *et al*. 2008; Dick, cited in Nowak *et al.* 2014). Additionally, Gross *et al.* (2005) reported on a pilot study showing that normal serum had no killing effect on *Neoparamoeba* spp. As such, it could be suggested that complement in the serum does not play a key role in providing protection against AGD.

In terms of anti-protease activity, trypsin inhibition varied little between the groups, with no significant effect of ploidy, infection, time or stock observed. A previous study investigating the effects of AGD on diploid and triploid Atlantic salmon also found no significant effect of ploidy on anti-proteases (Powell *et al*. 2008). However, due to the limited data regarding the involvement of anti-proteases in AGD and, more so, the lack of data concerning anti-protease activity in triploids, it is difficult to confirm whether the patterns observed in this study are expected. While anti-proteases are known to be involved in other parasitic infections such as *Cryptobia salmonista* and *Enteromyxum leei* (Alvarez-Pellitero, 2008; Sitja-Bobadilla *et al.* 2008), these are endoparasites and it could be suggested that as an ectoparasite, *N. perurans* does not elicit a response from anti-proteases in the serum.

In conclusion, this study found that the severity of AGD was not affected by ploidy and that both diploid and triploid exhibited a similar degree of pathology. However, considering the previously reported ploidy differences in AGD pathology, it is recommended that further studies be undertaken to fully elucidate ploidy differences in the development of AGD pathology. Complement and anti-protease activities were not influenced by ploidy or infection while lysozyme activity was significantly affected by ploidy but was not found out-with normal ranges. This could suggest that the serum innate immune response has a limited role in protecting against infection with *N. perurans*. Overall, the findings of this study would suggest that triploid salmon appear to have similar susceptibility to diploid siblings in response to disease challenge with *N. perurans* and provides a base for further research into the immune response of triploid Atlantic salmon.
